# Case Report: Abdominal Cocoon With Jejuno-Ileo-Colonic Fistula

**DOI:** 10.3389/fsurg.2022.856583

**Published:** 2022-04-28

**Authors:** Jian Jiao, Keshu Shan, Kun Xiao, Zhenjun Liu, Ronghua Zhang, Kangdi Dong, Jin Liu, Qiong Teng, Liang Shang, Leping Li

**Affiliations:** ^1^Shandong First Medical University, Jinan, China; ^2^Department of Gastrointestinal Surgery, Shandong Provincial Hospital Affiliated to Shandong First Medical University, Jinan, China; ^3^Department of Gastrointestinal Surgery, Feicheng Hospital Affiliated to Shandong First Medical University, FeiCheng People's Hospital, Feicheng, China; ^4^Department of Gastroenterology, Shandong Provincial Hospital Affiliated to Shandong First Medical University, Jinan, China; ^5^Cheeloo College of Medicine, Shandong University, Jinan, China

**Keywords:** abdominal cocoon, enteroenteric fistula, jejuno-ileo-colonic fistula, treatment, case report

## Abstract

**Introduction:**

Abdominal cocoon is a unique peritoneal disease that is frequently misdiagnosed. The occurrence of the abdominal cocoon with a jejuno-ileo-colonic fistula has not been previously reported.

**Case Presentation:**

We admitted a 41-year-old female patient with an abdominal cocoon and a jejuno-ileo-colonic fistula. She was admitted to our hospital for the following reasons: “the menstrual cycle is prolonged for half a year, and fatigue, palpitations, and shortness of breath for 2 months”. On the morning of the 4th day of admission, the patient experienced sudden, severe, and intolerable abdominal pain after defecating. An emergency abdominal CT examination revealed intestinal obstruction. Surgery was performed, and the small intestine and colon were observed to be conglutinated and twisted into a mass surrounded by a fibrous membrane, and an enteroenteric fistula was observed between the jejunum, ileum, and sigmoid colon. We successfully relieved the intestinal obstruction and performed adhesiolysis. The patient was discharged from our hospital on the 6th postoperative day, then she recovered and was discharged from Feicheng People's Hospital after another 11 days of conservative treatment, and she recovered well-during the 2-month follow-up period.

**Conclusion:**

Abdominal cocoon coexisting with a jejuno-ileo-colonic fistula is very rare. During the process of abdominal cocoon treatment, the patient's medical history should be understood in detail before the operation, and the abdominal organs should be carefully evaluated during the operation to avoid missed diagnoses.

## Introduction

An abdominal cocoon is a unique peritoneal disease discovered by Foo in 1978 and resembles a silkworm cocoon. The disease lacks typical clinical manifestations and is frequently misdiagnosed, leading to the administration of incorrect treatments (1–3). An intestinal fistula is an abnormal passage linking the bowel to other organs, the abdominal cavity, or areas outside the abdominal cavity, and it can arise due to inflammatory bowel disease, tumor complications, congenital diseases, abdominal surgery, or trauma (4, 5). However, no case report has been published on abdominal cocoons coexisting with enteroenteric fistulae. Here, we present a rare case of abdominal cocoon coexisting with a jejuno-ileo-colonic fistula, shedding further light on this disease.

## Case Presentation

A 41-year-old female was admitted to the Department of Endocrinology at our medical center for the following concerns: “the menstrual cycle is prolonged for half a year, and fatigue, palpitations, and shortness of breath for 2 months”. The patient reported a 2-year history of 10-min to 1-h abdominal pain and bloating episodes that were spontaneously resolved without treatment, and she had not been treated for the above symptoms. The physical examination revealed a flat abdomen, no tenderness or rebound tenderness in the abdomen, and bowel sounds present five times/min. The laboratory test results showed the following: a white blood cell count of 12.81^*^10^9^/l, a red blood cell count of 2.98^*^10^12^/l, a hemoglobin level of 48 g/l, and a C-reactive protein level of 9 mg/l. The gynecological ultrasound revealed a cervical cyst. The abdominal ultrasound showed that the patient had a fatty liver and a liver cyst. The patient had no history of abdominal surgery, inflammatory bowel disease, tuberculosis, or long-term medication use.

**Figure 1:**
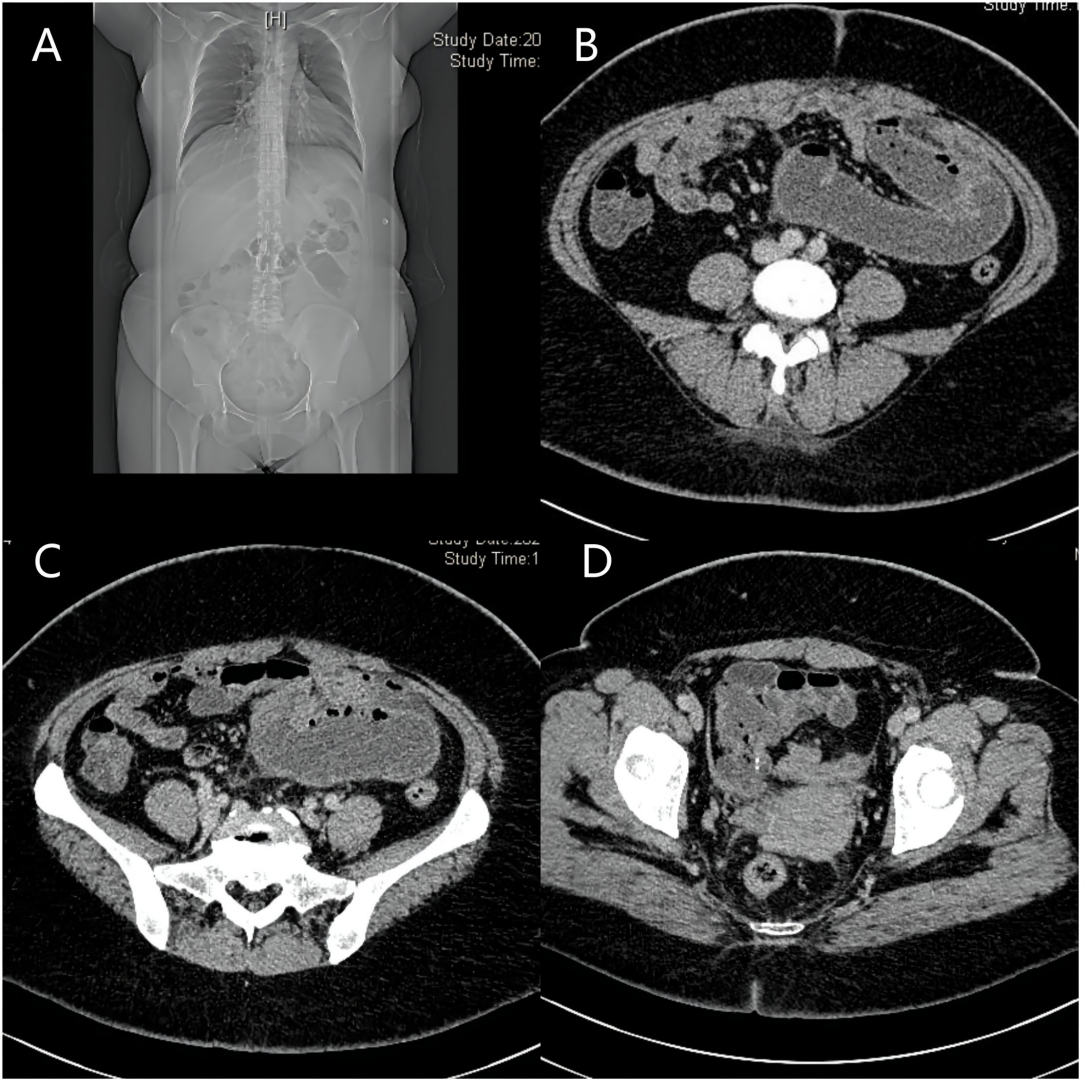
Images of abdomen CT. **(A–C)** show intestinal obstruction, which possibly caused by torsion of adhesions; **(D)**: part of the small intestine is wrapped by a fibrous tissue membrane and folded into a mass.

Iron and 4 U red blood cells were given symptomatically after admission to address the patient's severe anemia. On the morning of the 4th day of admission, the patient's condition changed, and she experienced sudden, severe, and intolerable abdominal pain after defecating. An emergency abdominal CT scan showed intestinal obstruction, possibly caused by the torsion of adhesions (Figure 1). Physical examination of the patient revealed abdominal distension, abdominal muscle tension, whole abdominal tenderness, rebound tenderness, and bowel sounds four times/ min.

**Figure 2:**
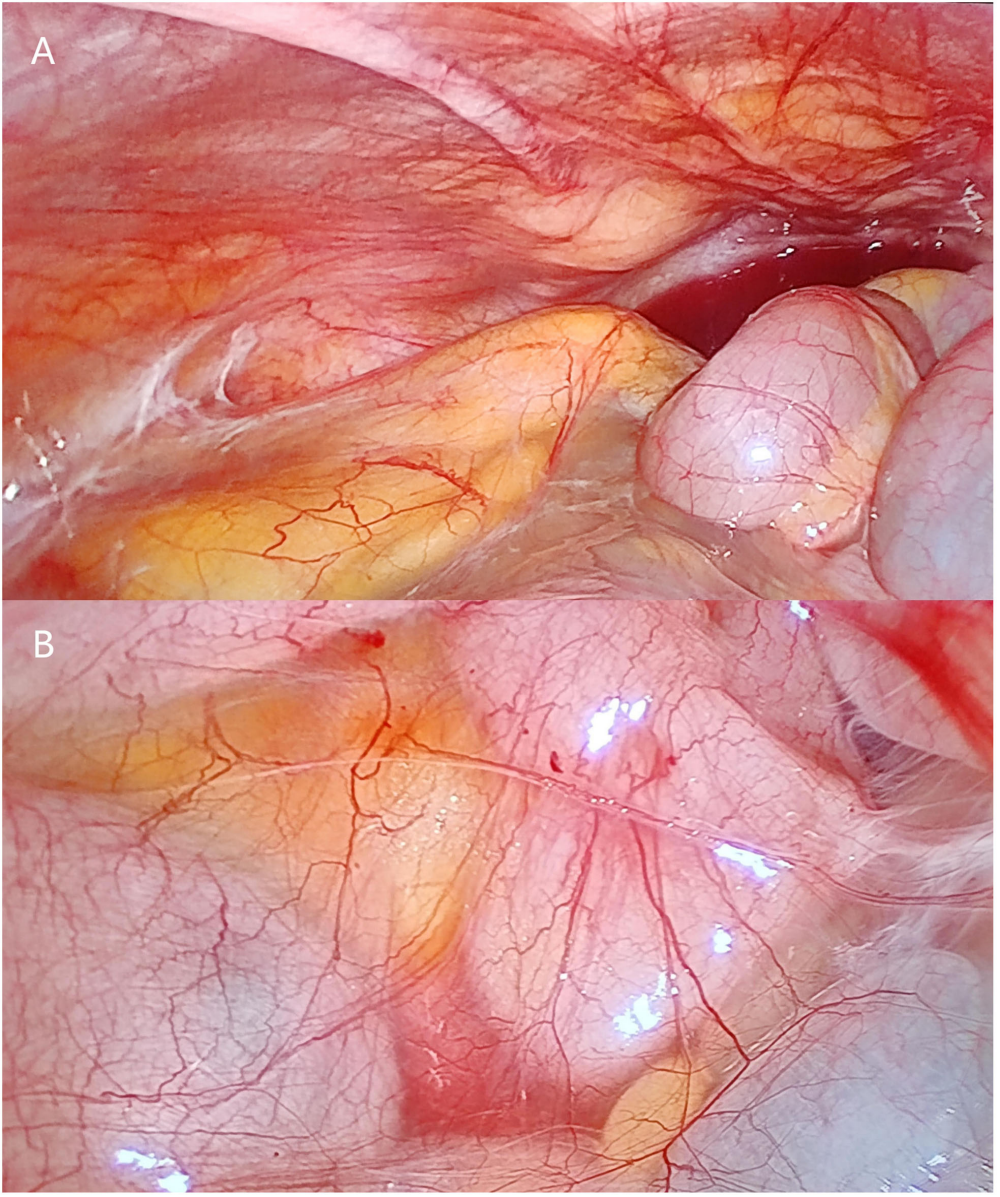
Photographs of laparoscopic surgery. **(A,B)** show that the small intestine is surrounded by fibrous membrane.

**Figure 3:**
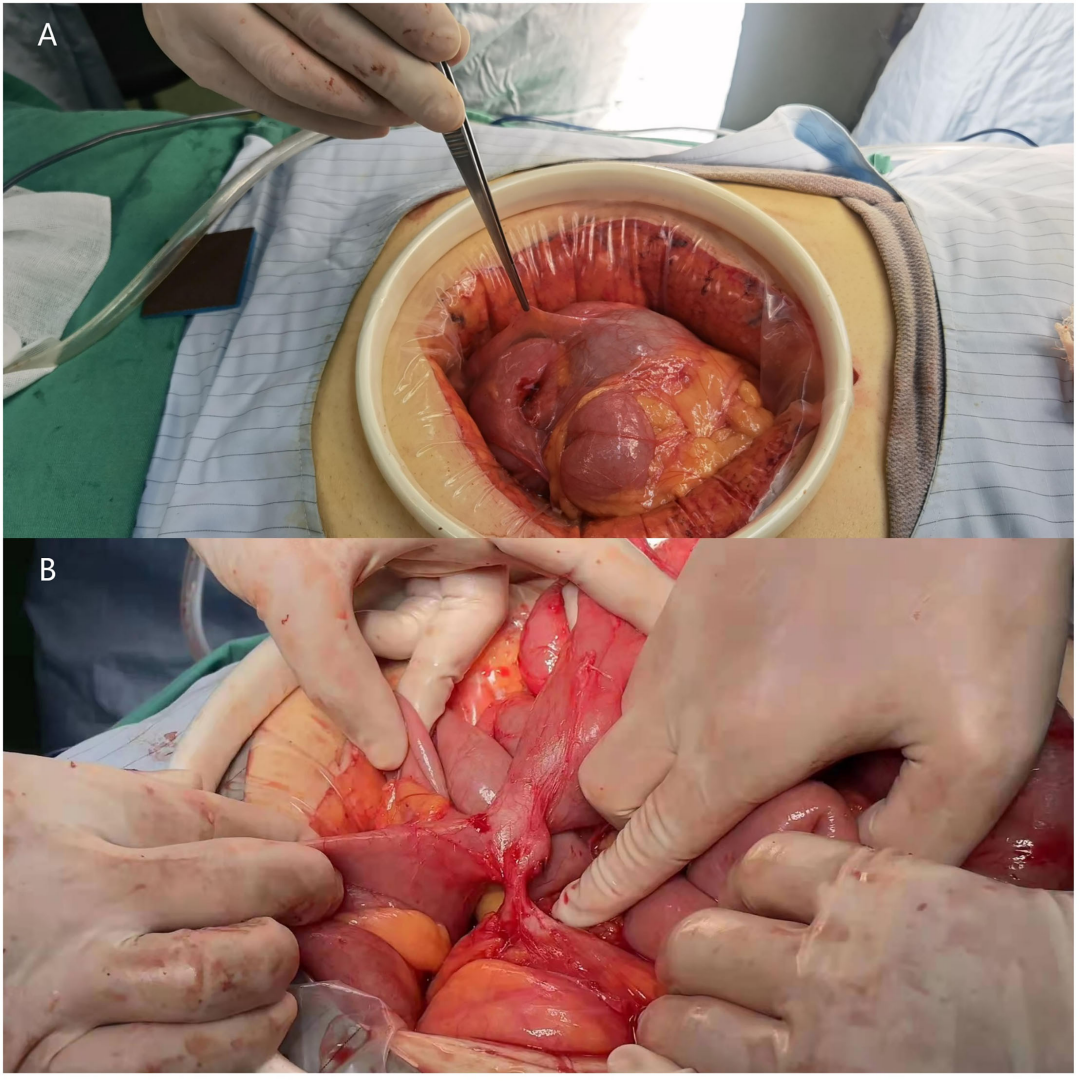
Photographs of the surgery scene. **(A)** Part of the small intestine is wrapped by a fibrous membrane and is folded into a mass. **(B)** jejuno-ileo-colonic fistula.

Emergency surgery to relieve the intestinal obstruction symptoms was performed after a discussion with the patient and family members. First, exploratory laparoscopic surgery was performed, and we observed that the small intestine and colon were conglutinated and twisted into a mass surrounded by a fibrous membrane (Figure 2). It was difficult to separate the adhesions under laparoscopy; thus, the decision to convert to open surgery was made, and the mid-abdomen was cut to allow access to the abdominal cavity. After separating the adhesions and freeing the bowel, a small intestine obstruction was found approximately 80 cm from the ligament of Treves, and an enteroenteric fistula was observed between the jejunum, ileum, and sigmoid colon (Figure 3).

Due to the obvious edema of the intestinal canal in the abdominal cavity, the enteroenteric fistula was relatively stable, and the patient had not undergone bowel preparation before surgery, after a detailed discussion about the surgical approach, we decided to focus on relieving the obstruction during the surgery to reduce the risk for postoperative anastomotic fistula and abdominal infection. The subsequent treatment of the jejuno-ileo-colonic fistula would be determined based on the patient's recovery and medical review. Ultimately, we successfully relieved the intestinal obstruction, performed adhesiolysis, and oriented the small bowel to its normal position. The intraoperative bleeding volume was approximately 50 ml, and 2 U of red blood cells and 200 ml of plasma were administered.

Anti-infection medication, gastrointestinal decompression treatment, antiacids, nutritional support, and iron supplementation were provided as symptomatic treatments after surgery. On the third postoperative day, the patient presented with anal exhaust and was able to consume water. On the fifth postoperative day, the patient defecated and began a complete liquid diet; on the same day, her laboratory test results showed a white blood cell count of 13.64^*^10^9^/l, a red blood cell count of 3.62^*^10^12^/l, and a hemoglobin level of 76 g/l. On the sixth postoperative day, the patient and her family expressed a strong desire to transfer to a local hospital closer to the patient's home for easier care. After we fully assessed the patient's condition and provided detailed information about the risks, the following future treatment plan was issued: (1) gradually return to a normal diet and continue anti-infection and nutritional support treatment, and (2) undergo dilatation and curettage on the first day of menstruation to explore the causes of the prolonged menstrual cycle. Finally, the patient was discharged from our hospital on the 6th postoperative. Then, after another 11 days of conservative treatment, she recovered and was discharged from Feicheng People's Hospital. We followed up with the patient by telephone. She reported a white blood cell count of 8^*^10^9^/l, and a hemoglobin level of 181 g/l on March 4, 2022 and she resumed a normal diet and daily exercise activities.

## Discussion

Abdominal cocoons, also known as sclerosing encapsulating peritonitis, are characterized by the wrapping of part or all of the small intestine with a fibrous tissue membrane that resembles a silkworm cocoon (1, 2, 6). An intestinal fistula is an abnormal passage between the bowel and abdominal organs or outside the abdominal cavity that usually occurs after abdominal surgery or trauma and can also arise due to inflammatory bowel disease, tumor complications, and congenital diseases (4, 5). Intestinal fistulae can be divided into enteroenteric fistula, enterobladder fistula, and enterovaginal fistula subtypes (4, 7). Abdominal cocoon complicated with an enteroenteric fistula has yet to be reported, and no study has been conducted on the relationship between the two diseases. We report one rare case of these conditions coexisting to improve understanding of abdominal cocoon.

Abdominal cocoon is rare, and most cases are diagnosed during exploratory laparotomy. It often presents with unexplained abdominal pain, abdominal distension, or intestinal obstruction (8–10). Typical abdominal CT scans show that the bowel is twisted and folded in the shape of an accordion, or a banana surrounded by a layer of dense linear membrane, and the mesenteric and blood vessels are often involved, twisted, and deformed (6, 11, 12). In this patient, the fibrous membrane wrapped around the small intestine and colon was thin, and she did not have a typical CT manifestation of the abdominal cocoon, which affected the accuracy of preoperative diagnosis.

The etiologies and pathogenesis of abdominal cocoons are unknown. Primary abdominal cocoon is more common in young women in tropical and subtropical areas and is often accompanied by congenital developmental defects, such as the absence or dysplasia of the greater omentum, cryptorchidism, infertility, congenital volvulus, mesenteric vascular malformation, or peritoneal variation. One possible mechanism is that the dysplastic peritoneum or greater omentum wraps part or all of the abdominal organs, affecting normal reproductive cell movement, and causing cryptorchidism or infertility (13–15). Another theory is that adolescent girls develop this condition secondary to viral infections and retrograde menstruation (1). The etiology of secondary AC is complex and is closely related to various suspected pathogenic factors, such as tuberculous peritonitis, a history of long-term peritoneal dialysis, the use of beta-blockers, a history of a gastrointestinal tumor, and a history of abdominal surgery (16–20). Long-term chronic inflammation secondary to the above factors causes the reduction of peritoneal mesothelial cells, a reduction in the activity of mesothelial transfer growth factor, the exudation of large levels of fibrin, and the ultimate development of the fibrous cocoon membrane, which hinders bowel movement (21, 22). This patient had no history of abdominal surgery, inflammatory bowel disease, tuberculosis, or long-term medication use; thus, this patient likely had a primary abdominal cocoon.

At present, surgery should be the first choice of treatment for patients with abdominal cocoon with severe acute abdomen symptoms, and the main goal of surgery is to separate and remove the fibrous membrane and loosen the adhesion between the intestines to allow movement and vitality (9, 23, 24). The membrane may wrap around the intestine and form a large mass, which is easily misdiagnosed as a gastrointestinal tumor. Abdominal cocoons should be studied further to avoid short bowel syndrome resulting from unnecessary resection of the intestine.

The treatment cycle of intestinal fistulae is long and difficult; it can cause multiple organ failures and even be life-threatening, and we should pay attention to this disease. Enteroenteric fistula is a special type of intestinal fistula (4), and its treatment should differ depending on the intestinal condition and the patient's condition (25). Patients with good systemic and intestinal conditions can directly undergo stage I intestinal resection and anastomosis; however, if the general condition is poor, enterostomy or drainage is also a feasible option. More importantly, enteral nutrition and parenteral nutrition are non-negligible treatments for delaying disease progression. This patient underwent emergency surgery for intestinal obstruction. Given that the intraoperative exploration showed obvious intestinal edema and that she did not undergo bowel preparation before the surgery, to reduce the risk of postoperative anastomotic fistula and abdominal infection, we only resolved the obstruction as we believed that it was more reasonable to treat the enteroenteric fistula at a later date based on the patient's recovery and medical review.

White blood cells participate in the body's defense system and reflect physiological changes in the body. Elevated white blood cell counts are mainly seen in infectious diseases (26). This patient had an intestinal obstruction, which resulted in increased inflammatory markers, including white blood cells. The patient's white blood cell count returned to normal with antibiotic treatment after surgery.

Anemia is a typical blood system disease involving a lower than normal red blood cell count (27). Anemia causes a series of symptoms, such as dizziness, fatigue, and a pale complexion. In severe cases, patients can experience symptoms such as breathing difficulties, a rapid heart rate, and shock. Etiological treatment and symptomatic treatment are generally conventional treatment methods. Our patient was initially hospitalized for severe anemia, and after red blood cell and iron supplementation, her hemoglobin levels returned to 181 g/l. To date, the patient has not undergone dilatation and curettage due to a marked improvement in her anemia symptoms. Although the etiology of the patient's prolonged menstrual cycle had not yet been found, combined with the existing examination results, we believe that the severe anemia may have been caused by the prolonged menstrual cycle. We will closely follow up with the patient to further determine the etiology of her prolonged menstrual cycle and monitor her recovery process.

## Conclusion

This article may represent the first report of an abdominal cocoon coexisting with a jejuno-ileo-colonic fistula. The above two diseases were not observed until the patient underwent surgery in this rare case. In conclusion, although the relationship between the two diseases is not yet clear, when treating patients with abdominal cocoon, we should understand the medical history in detail, consider whether the patient has a coexisting disease to avoid misdiagnosis and incorrect treatment, and provide better patient quality of life.

## Data Availability Statement

The raw data supporting the conclusions of this article will be made available by the authors, without undue reservation.

## Ethics Statement

The studies involving human participants were reviewed and approved by Ethics Committee of Shandong Provincial Hospital Affiliated to Shandong First Medical University. The patients/participants provided their written informed consent to participate in this study. Written informed consent was obtained from the individual(s) for the publication of any potentially identifiable images or data included in this article.

## Author Contributions

JJ, KS, and KX designed the study and wrote the original draft. JJ, KS, KX, and ZL participated in the treatment process of the patient. RZ, KD, JL, and QT collected the information and arranged to image. LS and LL wrote and revised the manuscript. All the authors contributed to the article and approved the submitted version.

## Funding

This paper was funded by the Science and Technology Innovation Development Program of Jinan (No. 2020019082); the Key Research and Development Program of Shandong Province (No. 2019JZZY010104); Special Foundation for Taishan Scholars Program of Shandong Province (No. ts20190978).

## Conflict of Interest

The authors declare that the research was conducted in the absence of any commercial or financial relationships that could be construed as a potential conflict of interest.

## Publisher's Note

All claims expressed in this article are solely those of the authors and do not necessarily represent those of their affiliated organizations, or those of the publisher, the editors and the reviewers. Any product that may be evaluated in this article, or claim that may be made by its manufacturer, is not guaranteed or endorsed by the publisher.
